# Association of sleep, screen time and physical activity with overweight and obesity in Mexico

**DOI:** 10.1007/s40519-019-00841-2

**Published:** 2019-12-31

**Authors:** Spyros Kolovos, Aura Cecilia Jimenez-Moreno, Rafael Pinedo-Villanueva, Sophie Cassidy, Gerardo A. Zavala

**Affiliations:** 1 Nuffield Department of Orthopaedics, Rheumatology and Musculoskeletal Sciences, University of Oxford, Old Rd, Oxford, OX3 7LD UK; 2 Wellcome Trust Centre for Mitochondrial Research, Institute of Neurosciences, Newcastle University, M4.026 Cookson Building, Frammlington Place, Newcastle upon Tyne, NE2 4HH UK; 3 MoveLab, Institute of Cellular Medicine, Newcastle University, 4th Floor, William Leech Building, Medical School, Framlington Place, Newcastle upon Tyne, NE2 4HH UK; 4 Department of Health Sciences, York University, Heslington, York YO10 5DD UK

**Keywords:** Obesity, Physical activity, Sleep, Lifestyle, ENSANUT 2016, Mexico

## Abstract

**Purpose:**

Approximately 70% of adults in Mexico are overweight or obese. Unhealthy lifestyle behaviors are also prevalent. We examined the association of three lifestyle behaviors with body mass index (BMI) categories in adults from Mexico.

**Methods:**

We used publicly available data from the ENSANUT 2016 survey (*n* = 6419). BMI was used to categorize participants. Differences in sleep duration, suffering from symptoms of insomnia, TV watching time, time in front of any screen, vigorous physical activity (yes vs no), moderate physical activity (> 30 min/day—yes vs. no) and walking (> 60 min/day—yes vs. no) were compared across BMI groups using adjusted linear and logistic regression analyses.

**Results:**

Thirty-nine percent of participants were overweight and 37% obese. Time in front of TV, in front of any screen, sleep duration and physical activity were significantly associated with overweight and obesity. Compared to normal weight participants, participants in the obese II category spend on average 0.60 h/day (95% CI 0.36–0.84, *p* = 0.001) and participants in the obese III category 0.54 h/day (95% CI 0.19–0.89, *p* < 0.001) more in front of any screen; participants in the obese II category reported 0.55 h/day less sleep (95% CI − 0.67 to − 0.43, *p* < 0.001); participants in the obese III category were less likely to engage in vigorous activity (OR = 0.60, 95% CI 0.43–0.84, *p* ≤ 0.003), or walking (OR = 0.65, 95% CI 0.49–0.88, *p* = 0.005).

**Conclusion:**

Screen time, sleeping hours, and physical activity were associated with overweight and obesity. However, these associations were not consistent across all BMI categories. Assuming established causal connections, overweight individuals and individuals with obesity would benefit from reduced screen time and engaging in moderate/vigorous physical activity.

**Level of evidence:**

Level III: observational case-control analytic study.

**Electronic supplementary material:**

The online version of this article (10.1007/s40519-019-00841-2) contains supplementary material, which is available to authorized users.

## Introduction

Overweight and obesity rates have increased during the last four decades globally [1]. More than half of the world’s population with obesity live in just ten countries, one of which is Mexico. Recent epidemiological studies have shown that approximately 70% of adults in Mexico are either overweight or obese [2, 3]. Obesity is a risk factor for a broad range of chronic diseases such as cardiovascular disorders and type II diabetes, and it has been associated with decreased life expectancy, high socio-economic costs, and impaired mental health [4, 5]. The Global Burden of Diseases, Injuries, and Risk Factors Study (GBD) identified diet as the leading risk category for premature death, and the second highest for disability-adjusted life-years (DALYs) worldwide [6]. The GBD Study further estimated that dietary risk factors were responsible for 11 million (22%) deaths and 255 million (16%) DALYs worldwide in 2017 [7].

Obesity is a multifactorial disease, often expressed as the result of a long-term energy imbalance between energy intake (diet) and energy expenditure (physical activity/sedentary periods) [8]. A reduction in activity levels, prolonged sedentary periods and poor sleep patterns have all been linked to an increased risk of obesity [9–11]. Watching television (TV) is also a lifestyle behavior associated with obesity, due to the lack of movement involved, the snacking related to TV viewing, or the combination of both [12, 13]. Sleep has been linked to energy metabolism and body mass index (BMI), with recent studies suggesting that too much or too little sleep is detrimental and may be associated with obesity [14–16]. Furthermore, sleep, physical activity, and screen time have been found to be concurrent in some cases, but in others their correlation was moderate to small and their associations were not statistically significant [17–19].

Several studies have characterized these lifestyle behaviors in Mexico [20–22]. One study found that the average time spent in front of the TV is 3 h per day country-wide for children between 10 and 18 years of age [21]; however, the respective information for adults is missing. Moreover, the overall average screen time is expected to be higher due to the increased use of mobile phones, tablets and computers [23]. Despite the World Health Organization (WHO) recommendations for physical activity (i.e., a minimum of 150 min of moderate or 75 min of vigorous physical activity per week [24]), in the last 12 years, there has been a significant reduction in physical activity levels in the Mexican population [20, 25]. Furthermore, daily sedentary time has increased by 8%, with an average increase of 18 min during a 9-year period (2006–2015) [22]. There is significant concern, moreover, that these behaviors seem to cluster in overweight and individuals with obesity [26], further increasing their risk of developing cardiovascular diseases, diabetes and other health problems [27, 28].

Although previous studies have examined the prevalence of overweight and obesity, and other studies have described lifestyle behaviors potentially linked to overweight and obesity, to the best of our knowledge, no studies have investigated the associations between all these factors in Mexico. Given the obesity epidemic in Mexico and the increase in unhealthy lifestyle behaviors, the aim of this study was to examine the association between modifiable lifestyle factors with overweight and obesity in the Mexican adult population. We addressed four specific research questions: (1) Is screen time associated with overweight and obesity? (2) Is sleep associated with overweight and obesity? (3) Is physical activity associated with overweight and obesity? and (4) Do lifestyle factors cluster together and is this cluster associated with overweight and obesity?

## Methods

### Data source and study population

The current study is an analysis of data collected with the 2016 Mexican National Health and Nutrition Survey (Spanish acronym: ENSANUT) [29]. ENSANUT is organized by the National Institute of Public Health and the Federal Ministry of Health in Mexico, and it provides information on the health and nutritional status of the Mexican population. ENSANUT is a probabilistic, multistage, stratified survey, representative of the Mexican population at national, state and municipality levels, with sufficient sampling power to differentiate between urban (≥ 2500 inhabitants) and rural (< 2500 inhabitants) areas. The sampling frame consisted of 11,000 households 6000 in urban and 5000 in urban regions. Three age groups were defined, including children (5–9 years), adolescents (10–19 years), and adults (more than 20 years), and, when available, one member of each age group was randomly selected from each household. Sampling weights were used to estimate nationally representative values. A more detailed description of the sampling procedures and survey methodology has been described elsewhere [30]. The ENSANUT 2016 survey was approved by the Research, Ethics and Biosafety Committees at the Mexican National Institute of Public Health, and it allows third parties to access and analyze data that will support standards of health and nutrition in the country. Written informed consent was obtained from all study participants and trained personnel administered all questionnaires and measurements face-to-face [30].

We used available data from 6419 adults, 20 years old and above, with complete records on BMI, physical activity and sleep duration. We used the STROBE checklist and guidelines for cross-sectional studies, which provide recommendations of reporting the study’s details for each section of the current manuscript [31]. The dataset used for the current study is publically available in the Encuesta Nacional de Salud y Nutrición 201 repository, https://ensanut.insp.mx/ensanut2016/descarga_bases.php.

### Variables

#### Anthropometric measurements

To calculate BMI, weight and height of participants were measured directly by trained personnel [32]. Participants were classified according to their BMI following the established WHO classification: underweight (BMI < 18.49), normal weight (BMI = 18.5–24.9), overweight (BMI = 25–29.9), obesity I (BMI 30–34.9), obesity II (BMI 35–39.9) and obesity III (BMI > 40) [33]. As an alternative indicator of obesity, waist circumference was measured at the nearest 0.1 cm at the minimum circumference between the bottom of the ribs and the top of the iliac using a flexible fiberglass anthropometric tape [34].

#### Screen time

Screen time was measured using a self-reported questionnaire, in which participants reported h/day spent on average during the last 30 days including weekdays and weekends (1) watching TV, and (2) in front of any screen (including TV, mobile phones, tablets and computers) [35]. The questionnaire has previously shown acceptable validity and reproducibility in a Mexican population [35].

#### Sleep

Sleep was assessed by means of: (1) sleep duration (participants were asked the average hours of sleep per day), and (2) suffering from symptoms of insomnia or not. To operationalize symptoms of insomnia participants were asked (1) if they had difficulties to fall asleep more than three times a week and (2) for how long they have been having this problem. If they had difficulties to fall asleep more than three times a week for the last three weeks, they were categorized as having symptoms of insomnia [36, 37].

#### Physical activity

The International Physical Activity Questionnaire (IPAQ) short form was used to measure physical activity. IPAQ estimates for each participant were provided by the ENSANUT 2016 and were established using the IPAQ data processing rules (i.e., truncating all physical activity estimates exceeding ‘3 h’ or ‘180 min’) [38]. The questionnaire has been previously validated in Mexican adults and has shown modest validity [39, 40]. Self-reported minutes per day were included for: (1) vigorous physical activity, (2) moderate physical activity, and (3) walking.

#### Clustering of lifestyle behaviors

We investigated the clustering of the lifestyle behaviors related to energy expenditure. To this aim, participants with no vigorous activity, poor sleep duration (< 7 or > 8 h/day) [41], and prolonged screen time (> 3 h/day) [26] were characterized as having an “unhealthy phenotype”. We used the cut-off of > 3 h/day to operationalize prolonged screen time because it has been used before in the literature [26] and it is above the previously reported average screen time for Mexico [21]. Thus, we created two clusters, one including participants meeting all three of the aforementioned criteria, and the other including the rest of the participants.

#### Covariates

A number of variables were extracted from the ENSANUT 2016 data set and included as covariates in the analyses: sex (women, men), age of participants (years), area of residence (rural, urban), previous diagnosis of diabetes (yes, no), and socioeconomic status (SES). Previous diagnosis of diabetes was self-reported, and it was included as covariate because previous studies have shown a very strong link to obesity and lifestyle behaviors [42–44]. SES was defined by an index, constructed by combining eight variables that assessed the household properties and available services including construction materials of the floor, ceiling, and walls; sleeping rooms; water accessibility; vehicle ownership; household goods (refrigerator, washing machine, microwave, stove, boiler); and electrical goods (TV, radio, telephone, and computer). SES index was divided into tertiles into the ENSANUT 2016 data set and used as a proxy for low, medium, and high SES.

### Statistical analysis

We extracted information for participants, selecting only those entries with complete data for the predefined variables for the study. Their clinical and demographic characteristics were detailed using descriptive statistics. Linear regression analyses were performed on dependent continuous variables and logistic regression on categorical variables. Dependent variables were each measure of screen time, sleep duration, and physical activity, with BMI categories as explanatory variables (i.e., underweight, normal weight, overweight, obese I, obese II, and obese III as dummy variables). Categorical dependent variables were symptoms of insomnia and having an unhealthy phenotype. Continuous dependent variables were sleep duration, TV time, and total screen time. The three physical activity variables were continuous (i.e. min/day) but they did not meet the assumption of linearity of residuals when included in the linear regression model and were, therefore, dichotomized using the median as cut-off. In particular, they were defined as ‘some vigorous physical activity’ if reported ≥ 1 min/day; ‘high moderate physical activity’ if reported > 30 min/day; and ‘high walking time’ if reported > 60 min/day. The choice of independent and dependent variables included in the regression models was based on existing evidence from studies examining the associations between BMI and lifestyle factors obesity [10].

As a second step, the variables sex, age, area of residence, SES, and history of diabetes were included as covariates in the adjusted models. These variables were tested as effect modifiers. Coefficients (*b*) and odds ratios (OR) were reported for the linear and logistic regression analyses, respectively, together with their 95% confidence intervals (95% CI). Because of the increased risk of type I error due to multiple statistical tests, we adjusted the *p* value using the Bonferroni correction. A *p* value of < 0.0072 was considered statistically significant for all statistical tests. The R “survey” package (version 3.5.2) was used to account for the complex survey design of ENSANUT, and the sampling weights were used in all the analyses.

We performed four sensitivity analyses to test the robustness of our findings. In the first sensitivity analysis, we used BMI but as a continuous variable, and in the second, we used waist circumference as a continuous variable instead of BMI as a proxy for overweight and obesity. To ensure a linear association in the latter, underweight participants were excluded from the analysis. These were performed on the basis of previous evidence that the association between obesity and other factors or diseases is sometimes influenced by the choice of anthropometric measure used to detect obesity [45]. The third sensitivity analysis, excluded participants in the underweight group, because of the very small sample size of this group, and merged all three obesity severity groups, because again obesity groups had relatively low sample sizes. Finally, we analyzed all the combinations of two clustered ‘unhealthy’ behaviors (i.e., high screen time and low physical activity; poor sleep duration and low physical activity; poor sleep duration and high screen time).

## Results

### Population characteristics

From the 8824 adults eligible in the ENSANUT 2016 data set, 6419 participants with complete information for the variables of interest were included in the analyses. The 2405 excluded participants did not have complete data for at least one of the variables we included in our analysis; 838 had no BMI or waist circumference data, 1053 no screen time, physical activity or sleep duration data, and 514 no sociodemographic data. There were no significant differences in demographic and clinical characteristics between the included and excluded participants.

The mean age of the included participants was 43 years (SD = 14) and 67% were women (Table 1). Half of the participants were living in urban areas and half in rural settings. From the sample, 1% was classified as underweight, 24% normal weight, 39% overweight, 25% as having obesity I, 8% as having obesity II and 4% as having obesity III (Table 1). The mean waist circumference was 95.9 cm (SD = 17.2), and 10% of the participants had history of diabetes. The detailed description of clinical and demographic characteristics separately for each sex can be found in the supplementary material (Table S1).

**Table 1:** Demographic and clinical characteristics of participants

	Underweight	Normal weight	Overweight	Obese I	Obese II	Obese III	Total
Participants, *n* (%)	65 (1%)	1519 (24%)	2479 (39%)	1579 (25%)	546 (8%)	231 (4%)	6419 (100%)
Women, *n* (%)	42 (65%)	903 (59%)	1596 (64%)	1146 (73%)	435 (80%)	183 (86%)	4305 (67%)
Age, mean (SD)	35 (15)	40 (15)	43 (13)	45 (13)	44 (12)	43 (12)	43 (14)
Rural area, *n* (%)	28 (43%)	788 (52%)	1279 (52%)	751 (48%)	258 (47%)	94 (44%)	3198 (50%)
Waist circumference, (cm) mean (SD)	69 (6)	83 (13)	94 (13)	102 (62)	112 (11)	125 (19)	96 (16)
Socioeconomic status							
Low, *n* (%)	26 (40%)	616 (40%)	846 (34%)	504 (32%)	148 (28%)	67 (31%)	2207 (34%)
Middle, *n* (%)	15 (23%)	506 (32%)	816 (33%)	540 (34%)	246 (45%)	76 (35%)	2199 (34%)
High, *n* (%)	24 (37%)	437 (28%)	794 (32%)	540 (34%)	145 (27%)	73 (33%)	2013 (31%)
Previous diagnosis of diabetes, *n* (%)	4 (6%)	86 (6%)	288 (12%)	183 (12%)	72 (13%)	29 (14)	662 (10%)

*SD* standard deviation

The mean total screen time for the whole sample was 2.36 (SD = 2.45) h/day and for TV 1.58 (SD = 1.49) h/day. The average sleep duration was 7.46 (SD = 1.43) h/day and 19% of the participants suffered from symptoms of insomnia. Finally, the average time for vigorous activity was 39 (SD = 66) min/day, for moderate activity 84 (SD = 74) min/day, and for walking 55 (SD = 58) min/day.

### Screen time

In the adjusted linear regression model, obese III category (*b* = 0.36, 95% CI 0.16–0.56, *p* < 0.001) was associated with higher TV time compared to normal weight category (Table 2). In contrast, no association was found for underweight, overweight, obese I and obese II categories. When total screen time was considered, overweight (*b* = 0.33, 95% CI 0.17–1.49, *p* < 0.001), obese I (*b* = 0.23, 95% CI 0.05–0.41, *p* = 0.006), obese II (*b* = 0.60, 95% CI 0.36–0.84, *p* = 0.001) and obese III categories (*b* = 0.54, 95% CI 0.19–0.89, *p* < 0.001) were associated with higher screen time per day as compared to normal weight category (Table 2). The covariates sex, age, area of residence, and SES were significantly associated with TV and screen time, with the strongest association found for SES (*b* = 0.96, 95% CI 0.88–1.04, *p* < 0.001).

**Table 2:** Adjusted linear model for screen time

	TV time (h/day)			Total screen time^a^ (h/day)		
	*b*	95% CI	*p*	*b*	95% CI	*p*
Underweight	0.22	− 0.13–0.57	0.213	0.62	0.03–1.21	0.040
Normal weight (reference category)	–	–	–	–	–	–
Overweight	− 0.01	− 0.09–0.07	0.818	0.33	0.17–0.49	< 0.001
Obesity I	0.10	0.00–0.20	0.052	0.23	0.05–0.41	0.006
Obesity II	0.06	− 0.08–0.20	0.401	0.60	0.36–0.84	0.001
Obesity III	0.36	0.16–0.56	< 0.001	0.54	0.19–0.89	< 0.001
Area (urban)	0.13	0.03–0.23	< 0.001	0.81	0.65–0.97	< 0.003
Age (years)	− 0.01	− 0.01 to − 0.01	< 0.001	− 0.07	− 0.07 to − 0.07	< 0.001
Sex (man)	0.13	0.05–0.21	< 0.001	0.46	0.34–0.58	< 0.001
History of diabetes (yes)	− 0.03	− 0.17–0.11	0.710	− 0.10	− 0.34–0.14	0.372
Socioeconomic status (tertiles)	0.27	0.23–0.31	< 0.01	0.96	0.88–1.04	< 0.001
Constant	1.05	0.85–1.25	< 0.01	2.05	1.72–2.38	< 0.001

*95% CI* 95% confidence interval, *b* beta coefficient*, p p* value

^a^Total screen time encompasses time in front of TV, mobile phones, tablets and computers

### Sleep

Compared to normal weight, overweight (*b *=− 0.18, 95% CI − 0.25 to − 0.09, *p* < 0.001), obese I (*b *=− 0.21, 95% CI − 0.31 to − 0.11, *p* = 0.003) and obese II categories (*b *=− 0.55, 95% CI − 0.67 to − 0.43, *p* < 0.001) were associated with fewer sleeping hours per day, as shown in adjusted regression model (Table 3). No statistical differences were found for the underweight and obese III categories as compared with the normal weight category. There were no differences in the odds of reporting symptoms of insomnia between normal weight participants and participants in the other BMI categories (Table 3). The covariates area of residence and SES included in the models were significantly associated with sleep duration and symptoms of insomnia.

**Table 3:** Adjusted linear and logistic models for sleep

	Sleep duration (h/day)			Symptoms of insomnia		
	*b*	95% CI	*p*	OR	95% CI	*p*
Underweight	0.18	− 0.13–0.49	0.25	1.39	0.84–2.30	0.931
Normal weight (reference category)	–	–	–	1.00	–	–
Overweight	− 0.17	− 0.25 to − 0.09	0.001	1.01	0.88–1.17	0.392
Obesity I	− 0.21	− 0.31 to -0.11	0.003	1.08	0.92–1.27	0.842
Obesity II	− 0.55	− 0.67 to − 0.43	< 0.001	1.17	0.94–1.46	0.151
Obesity III	− 0.17	− 0.37–0.03	0.091	1.25	0.92–1.70	0.852
Area (urban)	− 0.29	− 0.37 to − 0.21	< 0.001	1.35	1.20–1.52	0.007
Age (years)	− 0.01	− 0.06–0.04	0.423	1.01	1.00–1.01	0.062
Sex (man)	− 0.34	− 0.40 to − 0.28	< 0.001	0.62	0.54–0.70	0.018
History of diabetes (yes)	0.12	0.00–0.24	0.038	1.31	1.10–1.55	0.032
Socioeconomic status (tertiles)	− 0.14	− 0.18 to − 0.10	< 0.001	1.13	1.05–1.22	0.001
Constant	8.67	8.49–8.85	< 0.001	NA	NA	NA

*95% CI* 95% confidence interval, *b* beta coefficient, *OR* odds ratio, *p p* value

### Physical activity

Results from the adjusted logistic regression models indicated that participants in the obese II (OR = 0.72, 95% CI 0.58–0.90, *p* = 0.004), and obese III (OR = 0.60, 95% CI 0.43–0.84, *p* = 0.003) categories were less likely to engage in vigorous physical activity compared to normal weight participants (Table 4). Participants in the obese I category (OR = 0.84, 95% CI 0.73–0.96, *p* = 0.006) were less likely to engage in higher moderate physical activity compared to normal weight participants, whereas no differences were found for any of the other obesity categories. Participants in the obese III category (OR = 0.65, 95% CI 0.49–0.88, *p* = 0.005) were less likely to engage in higher walking time as compared to normal weight participants, whereas no differences were found for overweight and obesity I and II categories. All the covariates were related to vigorous physical activity; sex and area of residence were related to moderate physical activity; sex, history of diabetes, and area of residence were related to walking time (Table 4). From the included covariates, being a man showed the strongest association with physical activity (vigorous physical activity: OR = 4.92, 95% CI 4.42–5.49, *p*  ≤ 0.001; moderate physical activity: OR = 1.76, 95% CI 1.59–1.95, *p*  ≤ 0.001; walking: OR = 1.63, 95% CI 1.48–1.81, *p* ≤ 0.001).

**Table 4:** Adjusted logistic models for physical activity

	Vigorous physical activity (≥ 1 min/day)			Moderate physical activity (> 30 min/day)			Walking (> 60 min/day)		
	OR	95% CI	*p*	OR	95% CI	*p*	OR	95% CI	*p*
Underweight	0.65	0.39–1.11	0.122	1.39	0.85–2.28	0.194	0.89	0.55–1.44	0.626
Normal weight (reference category)	1.00	–	–	1.00	–	–	1.00	–	–
Overweight	0.98	0.86–1.12	0.791	0.90	0.79–1.02	0.063	1.01	0.90–1.14	0.846
Obesity I	0.85	0.76–0.97	0.009	0.84	0.73–0.96	0.006	0.93	0.81–1.06	0.281
Obesity II	0.72	0.58–0.90	0.004	1.00	0.82–1.20	0.844	0.89	0.74–1.08	0.247
Obesity III	0.60	0.43–0.84	0.003	1.08	0.82–1.43	0.571	0.65	0.49–0.88	0.005
Area (urban)	0.84	0.76–0.94	0.003	1.17	1.06–1.29	0.004	0.94	0.85–1.04	0.254
Age (years)	0.98	0.98–0.98	< 0.001	1.00	1.00–1.00	0.951	1.00	1.00–1.01	0.023
Sex (man)	4.92	4.42–5.49	< 0.001	1.76	1.59–1.95	< 0.001	1.63	1.48–1.81	< 0.001
History of diabetes (yes)	0.71	0.58–0.87	0.001	1.18	1.00–1.39	0.048	0.76	0.71–0.81	< 0.001
Socioeconomic status (tertiles)	0.92	0.86–0.99	0.017	0.96	0.90–1.02	0.225	0.72	0.61–0.85	< 0.001

*95% CI* 95% confidence interval, *b* beta coefficient, *OR* odds ratio, *p p* value

### Clustering of lifestyle factors

Approximately, 10% of the participants (*n* = 635) had an “unhealthy phenotype” (combined reported poor sleep duration, prolonged screen time, and no vigorous activity), of these 1% were in the underweight, 21% in the normal weight, 38% in the overweight, 27% in the obese I, 8% in the obese II and 5% in the obese III category. The adjusted logistic regression model showed that participants in the obese III category (OR = 1.79, 95% CI 1.18–2.72, *p* = 0.007) were more likely to have an unhealthy phenotype as compared with normal weight participants. No statistically significant differences were found for underweight, overweight, obese I, and obese II categories compared to the normal weight category. The covariates area of residence, age and SES were significantly associated with unhealthy phenotype (Table 5).

**Table 5:** Adjusted logistic models for unhealthy phenotype

	Unhealthy phenotype (*n* = 635)		
	OR	95% CI	*p*
Underweight	0.92	0.39–2.20	0.854
Normal weight (reference category)	1.00	–	–
Overweight	1.13	0.90–1.42	0.281
Obesity I	1.19	0.93–1.53	0.162
Obesity II	1.29	0.93–1.80	0.130
Obesity III	1.79	1.18–2.72	0.007
Area (urban)	1.74	1.44–2.10	< 0.001
Age (years)	0.98	0.98–0.99	< 0.001
Sex (man)	0.78	0.64–0.94	0.009
History of diabetes (yes)	1.15	0.85–1.54	0.360
Socioeconomic status (tertiles)	1.87	1.66–2.10	< 0.001

The unhealthy phenotype is defined as poor sleep duration, prolonged screen time, and no vigorous activity

*95% CI* 95% confidence interval, *OR* odds ratio, *p p* value

### Sensitivity analysis

The analysis using BMI as continuous explanatory variable showed results similar to the main analyses. The different adjusted regression models showed that higher BMI was associated with higher total screen time (*b *=0.12, 95% CI 0.07–0.17, *p* < 0.001) and TV time (*b *=0.14, 95% CI 0.06–0.23, *p* < 0.001), and lower vigorous physical activity (OR = 0.96, 95% CI 0.95–0.97, *p* = 0.001) (Table 6). Larger waist circumference was associated with higher total screen time (*b *=0.54 95% CI 0.39–0.68, *p* < 0.001) and TV time (*b *=0.63 95% CI 0.38–0.87, *p* < 0.001), poorer sleep duration (*b *= − 0.47 95% CI − 0.74 to − 0.21, *p* < 0.001), and vigorous (OR = 0.99, 95% CI 0.98–0.99, *p* < 0.001) physical activity (Table 6).

**Table 6:** Sensitivity analysis of the association between lifestyle behaviors with BMI as continuous score and waist circumference

	BMI (explanatory variable)			Waist circumference (cm) (explanatory variable)		
	*b* or OR^a^	95% CI	*p*	*b* or OR^a^	95% CI	*p*
TV (h/day)	0.14	0.06–0.23	< 0.001	0.63	0.38–0.87	< 0.001
Screen (h/day)	0.12	0.07–0.17	< 0.001	0.54	0.39–0.68	< 0.001
Sleep duration (h/day)	− 0.31	− 0.39 to − 0.24	0.821	− 0.47	− 0.74 to − 0.21	0.001
Symptoms of insomnia (yes)	1.00	0.99–1.00	0.231	1.00	1.00–1.00	0.081
Vigorous physical activity (higher)	0.96	0.95–0.97	0.002	0.99	0.98–0.99	< 0.001
Moderate physical activity (higher)	1.00	0.99–1.00	0.339	0.99	0.99–1.00	0.231
Walking (higher)	0.98	0.97–0.99	0.022	0.99	0.99–0.99	0.038

All the analyses were adjusted for age, sex, urban/rural, diabetes diagnose and socioeconomic status. Underweight participants were excluded from these analyses

^a^We reported beta coefficients for continuous outcomes and odd ratios (OR) for dichotomous outcomes

The results from the third sensitivity analysis, excluding underweight participants and merging the three obesity severity levels, were in accordance with the results from the main analyses. The three tables with the detailed results from the adjusted regression analyses for screen time, sleep, and physical activity can be found in the supplementary material (Tables S2, S3, and S4). The sensitivity analysis of the combination of clusters of ‘unhealthy’ behaviors is also in accordance with the main analysis of ‘unhealthy phenotype’. In all three regressions, participants in the obesity II and III categories were more likely to be in the cluster of two ‘unhealthy’ behaviors compared to participants in the normal weight category (Table S5).

## Discussion

This study investigated the association of different lifestyle factors with overweight and obesity in the Mexican population. The modifiable lifestyle factor most strongly associated with overweight and obesity was screen time (both for TV and total, including mobile phones, tablets, and computers). Sleep duration was associated with overweight, obesity I and obesity II but not with obesity III. Furthermore, the analysis of physical activity indicated that participants with obesity were less likely to engage in vigorous physical activity; whilst, participants in the obese I category were less likely to be involved in moderate physical activity and participants in the obese III category were also less likely to walk for more than 1 h a day, in all cases compared to normal weight participants. Combined reported poor sleep duration, prolonged screen time, and no vigorous activity were observed for small number of participants (10%), and participants in the obese III category were more likely to report this clustering.

Participants in the obese II and obese III categories reported more than half an hour extra screen time per day compared to normal weight participants. The respective association for TV time was not so strong, and for participants in the overweight and obese II categories, it was not statistically significant. The mean TV time found in our study was lower compared to the mean TV time previously reported in Mexican children [21]. Previous research has shown that overall screen time is a better proxy for sedentary behavior than just TV time [46]. Our results are in line with previous research indicating an association between overall screen time and obesity, demonstrating that reducing screen time could contribute to decreasing the prevalence of overweight and obesity [47, 48]. Thus, the reduction of screen time should be considered for weight management programs and it should also be modified for a non-sedentary activity to impact effectively on weight [13].

Participants in the overweight, obese I and obese II categories reported less sleeping time per day compared with normal weight participants. In particular, participants in the obese I category reported sleeping approximately half an hour less per day than those of normal weight. The small sample size for the obese III category and the respective limited statistical power to detect differences between the groups may explain the absence of association between sleeping hours and obese III category. Symptoms of insomnia were not related to overweight or obesity in our study. A number of causal pathways have linked short sleep duration with obesity, as demonstrated by experimental studies [49, 50]. For instance, sleep deprivation may stimulate appetite and increase caloric intake [51–53] due to a dysregulated production of hormones related to appetite [54, 55]. However, findings regarding the association between sleep disturbances and obesity are contradictory, since some studies showed a relationship but others did not confirm the association [56–58]. These results may be explained by the difficulty in defining problematic sleep patterns and associated limitations of self-reported outcomes as compared to objective measures. The ideal sleep duration varies amongst people and it remains subjective, especially for insomnia as it is a self-reported group of symptoms related to sleep behaviors [56]. The ideal method to corroborate these findings should use a more objective approach, like the use of activity monitors (e.g., accelerometers) or sleep tracking technology.

This study identified that participants with obesity from all three categories were less likely to engage in vigorous physical activity as compared to normal weight participants. However, these associations did not hold for moderate physical activity, where the only statistically significant difference was found for participants in the obese I category compared to normal weight participants. Similarly, only obese III participants were less likely to engage in at least an hour of walking time per day. A previous study using data from the ENSANUT 2006 and 2012 in Mexico found only a small difference between BMI categories and reported moderate-to-vigorous physical activity minutes per week [20]. For example, overweight participants engaged in four more minutes of physical activity per week when compared to the normal weight group based on data from the ENSANUT 2006, and 3 min less as indicated by ENSANUT 2012. In both surveys, participants in the obese group engaged in lower physical activity (i.e., 17–25 min less per week) compared to the normal weight group. Therefore, it seems that the association of physical activity and overweight and obesity in the Mexican population is not conclusive and more research is required. Future studies looking into this association should use objective devices, such as accelerometers, to measure physical activity [59, 60].

In this study, screen time was associated with overweight and obesity, whereas the association between physical activity and obesity appears more complex. This may be explained by the growing evidence that sedentary behavior, for which screen time can be considered a proxy, is a distinct risk factor for various conditions, including obesity, and it may be independent of physical activity [10, 47]. It is possible that the public is more informed about the positive impact of physical activity and less informed about the negative impact of sedentary behavior, which may be reflected by the results from the screen time analysis. Thus, the findings from this study could be used as evidence to update public health guidelines.

Participants in the obese III category had 1.79 times higher odds to have an “unhealthy phenotype” compared to normal weight participants. About 10% of the participants were included in the “unhealthy phenotype” group (i.e., reported simultaneously poor sleep duration, high screen time, and no vigorous activity). In line with our findings, a previous study found that 10% of participants with comorbid type 2 diabetes and cardiovascular disease (CVD) reported poor sleep duration, high TV time, and low physical activity clustered together [26]. This suggests that even though the clustering of negative lifestyle factors is not very prevalent, it does occur in some individuals, for whom the risk for various health conditions may be increased. These individuals should be targeted as priority on health intervention programs.

From the covariates included in the analyses, sex, the area of residence and SES were consistently associated with the different lifestyle factors. For instance, men reported more screen time and higher odds of engaging in vigorous physical activity compared to women. Overall, it appears that these sociodemographic factors influence the association between BMI and the modifiable behaviors. Reasons behind these differences could inform health prevention plans in low- and middle-income countries. These cultural and sex differences should be addressed when designing intervention programs to improve lifestyle behaviors and these should be designed population specific [61].

Results from the sensitivity analyses were, in general, in accordance with the results from the main analyses. The two analyses including BMI and WC as continuous outcome variable yielded similar results. The only difference was that WC was associated with sleep duration but BMI was not. This may be an indication that the association between obesity and lifestyle factors is influenced by the measure used to operationalize obesity, which has been reported before in similar studies [5].

### Strengths and limitations

This study was undertaken based on a large-scale survey representative of the Mexican population, thereby signaling that its statistically significant findings are widely applicable in the wide Mexican context. We conducted sensitivity analyses to examine if the operationalization of overweight and obesity influenced our findings and demonstrated that the results were similar across all analyses. Furthermore, obesity was measured by trained personnel using objective measures and was not self-reported by the participants [62].

However, our analyses faced limitations. The variables included as outcomes were reported by the participants themselves and, therefore, are subject to recall bias [63], which may have resulted for instance in an underestimation of the percentage of participants with unhealthy phenotype and an overestimation of the reported physical activity [64]. Moreover, the study has a cross-sectional design that does not allow to draw any conclusions on the direction of causality between BMI categories and lifestyle factors [65]. The WHO recommends a minimum of 150 min of moderate physical activity or 75 min of vigorous physical activity per week, but we could not use this cut-off in our study due to the very high percentage of participants reporting values exceeding those guidelines [24]. It has been reported that some participants over-report their physical activity levels in these types of surveys [66], and for this reason we used the median of each physical activity variable as the cut-off in corresponding analyses. Finally, the criteria for diagnosis of insomnia based on the Diagnostic and Statistical Manual of Mental Disorders 5th edition (DSM-5) regard the past 3 months, whereas the question in the ENSANUT survey regarded the past 3 weeks. The question about duration of symptoms can be amended in future surveys to resemble more the DSM criteria for each disorder.

## Conclusion

This is the first study to examine different modifiable lifestyle factors associated with BMI categories in Mexico, a highly relevant undertaking considering the high rates of overweight and obesity in the country’s adult population. Screen time, sleeping hours, and physical activity were found to be associated with obesity. However, these associations were not consistent across all obesity levels. Unhealthy lifestyle factors seem to cluster together for a few individuals, and the risk of this clustering may be increased for obese individuals. Given the high prevalence of obesity and unhealthy behaviors in Mexico and the limited effectiveness of current strategies to tackle obesity, multi-disciplinary lifestyle interventions should be explored to tackle the obesity epidemic in the country. Assuming established causal connections, individuals with obesity would benefit from improved sleep, reduced screen time and engaging in vigorous physical activity.

## What is already known on this subject?

Approximately, 70% of adults in Mexico are overweight or obese. Unhealthy lifestyle behaviors, such as low physical activity, poor sleep duration, and prolonged screen time, are also prevalent. However, no studies have investigated the associations between obesity and unhealthy lifestyle behaviors in Mexico.

## What does this study add?

Screen time, sleeping hours, and physical activity were found to be associated with obesity; although, these associations were not consistent across all obesity levels.

## Electronic supplementary material

Below is the link to the electronic supplementary material.

Supplementary material 1 (DOCX 28 kb)
