# Supplementary material for: Association of sleep, screen time and physical activity with overweight and obesity in Mexico
Source: Eat Weight Disord. 2019 Dec 31;26(1):169–79. doi: 10.1007/s40519-019-00841-2 (PMC7895770; doi:10.1007/s40519-019-00841-2)
Supplement: Supplementary file 1 — Supplementary material 1 (DOCX 28 kb) [file 40519_2019_841_MOESM1_ESM.docx]

**Supplementary material**

| **Table S1**. Demographic and clinical characteristics of the population by sex | | | |
| --- | --- | --- | --- |
|  | **Female (4,217)** | **Male (2,202)** | **Overall (6,419)** |
| Body Mass Index(units) mean (SD) | 29.31 (5.59) | 27.79 (4.95) | 28.79 (5.43) |
| Waist circumference (cm) mean (SD) | 95.6 (17.35) | 96.7 (17.15) | 95.9 (17.2) |
| Age mean (SD) | 40.88 (11.97) | 41.48(12.65) | 3246(50.2) |
| Rural area n (%) | 2075 (51.4) | 1054 (47.9) | 43 (13) |
| Socioeconomic status |  |  |  |
| Low n (%) | 1383 (32.4) | 789(36.2) | 2172 (34.0) |
| Middle n (%) | 1425 (34.6) | 727 (33.0) | 2152 (33.7) |
| High n (%) | 1409 (33.0) | 677 (30.7) | 2086 (32.2) |
| Sleep duration (hours) | 7.53(1.42) | 7.27(1.39 | 7.46(1.43) |
| Screen time (hours/day) | 2.31(2.47) | 2.68(2.57) | 2.36(2.45) |
| TV time (hours/day) | 1.53(1.48) | 1.72(1.51) | 1.58(1.49) |
| Vigorous physical activity | 21.35(48.36) | 82.69(80.62) | 39.35 (66.35) |
| Moderate physical activity | 92.32 (73.06) | 69.17(72.72) | 84.22 (74.25) |
| Walking | 49.17 (53.86) | 68.99 (65.32) | 55.12(58.21) |

The tables S2, S3, and S4 present the results from the third sensitivity analysis, in which underweight participants were excluded and the three obesity severity groups were merged.

| **Table S2.** Adjusted linear model for screen time | | | | | | |
| --- | --- | --- | --- | --- | --- | --- |
|  | **TV time (hours/day)** | | | **Total screen time (hours/day)** | | |
|  | **b** | **95% CI** | ***p*** | **b** | **95% CI** | ***p*** |
| Normal weight (reference category) | - | - | - | - | - | - |
| Overweight | -0,02 | -0,11 to 0,07 | 0,68 | 0,30 | 0,16 to 0,45 | <0,01 |
| Obesity | 0,10 | 0,01 to 0,19 | 0,02 | 0,32 | 0,17 to 0,48 | <0,01 |
| Area (urban) | 0,14 | 0,05 to 0,23 | <0,01 | 0,83 | 0,67 to 0,98 | <0,01 |
| Age (years) | 0,01 | -0,01 to 0 | <0,01 | 0,07 | -0,08 to -0,07 | <0,01 |
| Sex (man) | 0,13 | 0,06 to 0,2 | <0,01 | 0,47 | 0,35 to 0,58 | <0,01 |
| History of diabetes (yes) | 0,02 | -0,15 to 0,11 | 0,75 | -0,09 | -0,32 to 0,13 | 0,42 |
| Socioeconomic status (tertiles) | 0,27 | 0,22 to 0,32 | <0,01 | 0,96 | 0,88 to 1,04 | <0,01 |
| Constant | 1,06 | 0,87 to 1,26 | <0,01 | 2,09 | 1,76 to 2,42 | <0,01 |

*Notes: 95% CI = 95% confidence interval; b = beta coefficient; p = p-value*

| **Table S3.** Adjusted linear and logistic models for sleep | | | | | | |
| --- | --- | --- | --- | --- | --- | --- |
|  | **Sleep duration (hours/day)** | | | **Symptoms of insomnia** | | |
|  | **b** | **95% CI** | ***p*** | **OR** | **95% CI** | ***p*** |
| Normal weight (reference category) | - | - | - | 1.00 | - | - |
| Overweight | -0,17 | -0,25 to -0,09 | <0,01 | 1 | 0,87 to 1,15 | 0,97 |
| Obesity | -0,29 | -0,37 to -0,21 | <0,01 | 1,1 | 0,95 to 1,27 | 0,19 |
| Age (years) | -0,31 | -0,38 to -0,22 | <0,01 | 1,36 | 1,2 to 1,53 | <0,01 |
| Sex (man) | 0.01 | -0,01 to 0 | <0,01 | 1,01 | 1 to 1,01 | <0,01 |
| History of diabetes (yes) | -0,35 | -0,41 to -0,29 | <0,01 | 0,61 | 0,54 to 0,7 | <0,01 |
| Socioeconomic status (tertiles) | 0,12 | 0,01 to 0,24 | 0,04 | 1,31 | 1,11 to 1,55 | <0,01 |
| Constant | -0,14 | -0,18 to -0,09 | <0,01 | 1,13 | 1,05 to 1,22 | <0,01 |
| *Notes: 95% CI = 95% confidence interval; b = beta coefficient; OR= Odds ratio; p = p-value* | | | | | | |

| **Table S4.** Adjusted logistic models for physical activity | | | | | | | | | |
| --- | --- | --- | --- | --- | --- | --- | --- | --- | --- |
|  | **Vigorous physical activity**  **(≥1 minutes/day)** | | | **Moderate physical activity**  **(>30 minutes/day)** | | | **Walking**  **(>60 minutes/day)** | | |
|  | **OR** | **95% CI** | **p** | **OR** | **95% CI** | **p** | **OR** | **95% CI** | **p** |
| Normal weight (reference category) |  |  |  |  |  |  |  |  |  |
| Overweight | 1,00 | 0,88 to 1,14 | 0,98 | 0.90 | 0.79 to 1.02 | 0.09 | 1,02 | 0,9 to 1,15 | 0,78 |
| Obesity | 0,87 | 0,76 to 1 | 0,04 | 0.86 | 0.74 to 0.97 | 0.03 | 0,9 | 0,79 to 1,02 | 0,09 |
| Area (urban) | 0,84 | 0,75 to 0,94 | <0.01 | 1.17 | 1.06 to 1.29 | <0.01 | 0,94 | 0,85 to 1,04 | 0,23 |
| Age (years) | 0,98 | 0,98 to 0,98 | <0.01 | 1.00 | 1.00 to 1.00 | 0.95 | 1 | 1 to 1,01 | 0,02 |
| Sex (man) | 4,95 | 4,45 to 5,52 | <0.01 | 1.74 | 1.58 to 1.95 | 0.01 | 1,64 | 1,49 to 1,82 | <0,01 |
| History of diabetes (yes) | 0,71 | 0,58 to 0,86 | <0.01 | 1.17 | 1.00 to 1.39 | 0.05 | 0,72 | 0,6 to 0,85 | <0,01 |
| Socioeconomic status (tertiles) | 0,92 | 0,86 to 0,99 | 0,02 | 0.96 | 0.90 to 1.02 | 0.20 | 0,76 | 0,71 to 0,81 | <0,01 |
| *Notes: 95% CI = 95% confidence interval; OR= Odds ratio; p = p-value* | | | | | | | | | |

| **Table S5.** Combinations of clusters of two ‘unhealthy’ behaviors | | | | | | | | | |
| --- | --- | --- | --- | --- | --- | --- | --- | --- | --- |
|  | **Increased screen time and low physical activity**  **(n= 1,342)** | | | **Poor sleep and low physical activity**  **(n=800)** | | | **Poor sleep and increased screen time**  **(n=277)** | | |
|  | **OR** | **95% CI** | **p** | **OR** | **95% CI** | **p** | **OR** | **95% CI** | **p** |
| Underweight | 1.16 | 0.49 to 2.77 | 0.736 | 1.10 | 0.38 to 3.2 | 0.859 | 0.13 | 0.01 to 1.21 | 0.073 |
| Normal weight (reference category) | 1.04 | 0.83 to 1.32 | 0.721 | 0.97 | 0.75 to 1.27 | 0.867 | 1.28 | 0.91 to 1.81 | 0.151 |
| Overweight | 1.04 | 0.8 to 1.37 | 0.745 | 1.09 | 0.82 to 1.45 | 0.562 | 1.52 | 1.04 to 2.24 | 0.031 |
| Obesity I | 1.92 | 1.32 to 2.81 | 0.001 | 1.35 | 0.88 to 2.07 | 0.163 | 1.29 | 0.71 to 2.38 | 0.401 |
| Obesity II | 3.07 | 1.65 to 5.72 | <0.001 | 2.253 | 1.16 to 4.36 | 0.016 | 5.48 | 2.36 to 2.71 | <0.001 |
| Obesity III | 2.42 | 2 to 2.94 | <0.001 | 1.57 | 1.27 to 1.95 | <0.001 | 2.82 | 2.14 to 3.74 | <0.001 |
| Area (urban) | 0.97 | 0.96 to 0.98 | <0.001 | 1.02 | 1.02 to 1.04 | <0.001 | 0.95 | 0.94 to 0.97 | <0.001 |
| Age (years) | 0.34 | 0.28 to 0.42 | <0.001 | 0.23 | 0.19 to 0.3 | <0.001 | 2.03 | 1.54 to 2.68 | <0.001 |
| Sex (man) | 1.68 | 1.19 to 2.38 | 0.003 | 1.41 | 0.99 to 1.99 | 0.055 | 1.29 | 0.77 to 2.19 | 0.332 |
| History of diabetes (yes) | 2.01 | 1.78 to 2.28 | <0.001 | 1.25 | 1.09 to 1.44 | 0.001 | 2.35 | 1.97 to 2.81 | <0.001 |
| Socioeconomic status (tertiles) | 1.16 | 0.49 to 2.77 | 0.736 | 1.10 | 0.38 to 3.2 | 0.859 | 0.13 | 0.01 to 1.21 | 0.073 |
| *Notes: 95% CI = 95% confidence interval; OR= Odds ratio; p = p-value* | | | | | | | | | |
